# Coagulation profile in severe COVID-19 patients: what do we know so far?

**DOI:** 10.5935/0103-507X.20200031

**Published:** 2020

**Authors:** Felicio Savioli, Leonardo Lima Rocha

**Affiliations:** 1 Skaggs School of Pharmacy, University of California San Diego - Califórnia, Estados Unidos.; 2 Medicina Intensiva, Hospital Alemão Oswaldo Cruz - São Paulo (SP), Brasil.

Severe coronavirus disease 2019 (COVID-19) patients may present with single organ failure, but some of them progress to more systemic and multiple organ dysfunctions. One of the most significant markers of poor prognosis in those patients is the development of coagulopathy. These critically ill patients may have abnormal coagulation parameters, which may lead to hypercoagulability and increase the risk of thromboembolic events. Also, severe COVID-19 patients have been associated with disseminated intravascular coagulation (DIC) and increased risk of death.^(1)^

In a recent publication, Tang et al.^(2)^ retrospectively assessed the coagulation profile of 183 new coronavirus pneumonia patients (both survivors and non-survivors). Non-survivors had higher levels of D-dimer, fibrin degradation products, and longer prothrombin time on admission as compared to survivors. Moreover, the authors found that 71.4% of non-survivors had criteria for DIC. Also, non-survivors had lower levels of fibrinogen and antithrombin (AT) late during the hospitalization.

In another retrospective study, Llitjos et al.^(3)^ screened 26 severe COVID-19 patients for venous thromboembolism in two intensive care units (ICU), using a complete duplex ultrasound from the thigh to the ankle. Despite all patients were submitted to anticoagulation, either prophylactic or therapeutic, the incidence of deep-vein thrombosis was of 53,8%, and pulmonary embolism was identified in 23% of the cases. Additionally, the authors found venous thromboembolism in 56%, and pulmonary embolism in 33% of the patients undergoing therapeutic anticoagulation upon admission to the ICU. Although fibrinogen levels, D-dimer, and platelet counts were measured in these patients, AT levels were not assessed.

A prospective observational study analyzed the coagulation profile of severe COVID-19 patients and assessed the improvement of hypercoagulable states following the implementation of a thromboprophylaxis protocol. In this study, Ranucci et al.^(4)^ reported on the coagulation profile of 16 patients. Upon admission to the ICU, all the patients were on a prophylactic-dose low molecular weight heparin (LMWH). Based on their protocol, the LMWH dose could be increased, AT levels would be considered correct when found below 70%, and/or clopidogrel could be given when platelet counts were above 400,000/µL. Of note, 25% of these patients had low AT levels. After adjusting the anticoagulation dose based on standard and viscoelastic coagulation tests suggesting hypercoagulability, the authors concluded that the coagulation profile trended to normalization.

Furthermore, Panigada et al.^(5)^ evaluated 24 intubated COVID-19 ICU patients and assessed their thromboelastographic and standard coagulation profiles. The authors observed that K and R values were 50% and 83%, respectively, shorter in COVID-19 patients. Also, the K angle and maximum amplitude values were respectively 72% and 83% higher in these patients, suggesting a hypercoagulable state. Besides, fibrinogen levels, D-dimer, Factor VIII, and Von Willebrand factor antigen were increased. On the other hand, AT levels were reduced.

Disseminated intravascular coagulation in sepsis is an acute inflammatory response leading to endothelial and tissue injury, and ultimately to multiorgan failure. Therefore, the identification of patients with coagulopathy is of paramount importance to decide either or not to start anticoagulation therapy. The efficacy of anticoagulant therapy for sepsis‐associated DIC remains controversial. According to the International Society of Thrombosis and Haemostasis (ISTH) guidance, LMWH are considered superior to unfractionated heparin for the treatment of thrombosis and prophylaxis for venous thrombosis, nevertheless not supported by high‐quality evidence.^(6)^

In a retrospective study, Tang et al.^(7)^ assessed the coagulation profile and outcomes of 449 severe COVID-19 patients. Also, the 28-day-mortality between heparin users and non-users were analyzed. Ninety-nine patients (22%) received heparin for at least seven days and, of those, 97 (21,6%) met the criteria for sepsis-induced coagulopathy (SIC). The authors evaluated the association between heparin therapy and outcomes according to the SIC score. They observed that heparin therapy was associated with lower mortality rates in patients with SIC scores ≥ 4, however, this was not observed in patients with SIC scores ≤ 4. Moreover, the authors observed a 20% reduction in mortality among heparin users when their D-dimer exceeded 3µg/mL.

It should be mentioned that the D-dimer test is restricted to the preclusion of thrombosis, as it has poor specificity and high sensitivity, being, therefore, more likely to provide false-positive results.^(8)^ Moreover, a negative D-dimer test could be used for decision-making on anticoagulation therapy discontinuation in venous thromboembolism patients, however, this approach remains arguable and additional studies are required to validate this proposal.^(9)^

Disseminated intravascular coagulation is an acquired syndrome characterized by intravascular coagulation activation arising from different causes. It can both originate from and cause damage to the microvasculature, that, if sufﬁciently severe, can cause organ dysfunction. Although the cause may differ, the systemic coagulation activation is a feature commonly seen in DIC.^(10)^

The diagnosis of DIC in critically ill patients is made by standard coagulation tests, which can only determine a 5% of thrombin generation and are not suitable to discriminate patients with prothrombotic states from those with increased risk of bleeding. On the other hand, viscoelastic tests assess the whole clot formation and degradation, being able to characterize whether a patient has an hypocoagulable or hypercoagulable state, making them good DIC management tools.^(11)^

The coagulation profile in severe cases of COVID-19 is complex and may have different presentations. Many of these patients may present with a hypercoagulable state due to inflammatory response, comorbidities, immobilization, the use of mechanical ventilation, and/or *extracorporeal membrane oxygenation*. A key point in these patients is to define the best testing strategies to assess the risk of a thromboembolic event.

Although clinical studies suggest the occurrence of prothrombotic state among severe COVID-19 patients, so far there data are insufficient to support the systematic use of therapeutic anticoagulation in such patients. For the present time, we suggest that additional studies should be performed focusing on coagulation testing, to provide a better understanding of the coagulation profile in severe COVID-19 patients, improve the assessment of coagulation-related risks, and support decisions on the appropriated anticoagulation therapy.
